# Spontaneous pneumothorax during nintedanib therapy in patients with systemic sclerosis‐associated interstitial lung disease

**DOI:** 10.1002/rcr2.716

**Published:** 2021-01-27

**Authors:** Toshiyuki Sumi, Hirofumi Uehara, Makoto Tada, Yoshiko Keira, Koki Kamada, Naoki Shijubou, Yuichi Yamada, Hisashi Nakata, Yuji Mori, Hirofumi Chiba

**Affiliations:** ^1^ Department of Pulmonary Medicine Hakodate Goryoukaku Hospital Hakodate Japan; ^2^ Department of Respiratory Medicine and Allergology Sapporo Medical University School of Medicine Sapporo Japan; ^3^ Department of Thoracic Surgery Hakodate Goryoukaku Hospital Hakodate Japan; ^4^ Department of Surgical Pathology Hakodate Goryoukaku Hospital Hakodate Japan

**Keywords:** Interstitial lung disease, nintedanib, pneumothorax, SSc‐ILD, systemic sclerosis

## Abstract

Interstitial lung disease (ILD) is a common complication of systemic sclerosis (SSc). Nintedanib, an antifibrotic drug, has recently been approved for treating SSc‐ILD. Although there have been no reports suggesting the development of pneumothorax with nintedanib use, its safety in patients with impaired lung function is unclear. We observed the development of refractory spontaneous pneumothorax during nintedanib therapy in two patients with SSc‐ILD and impaired lung function. Nintedanib use for SSc‐ILD, an extensive disease, may therefore increase the risk of pneumothorax. In addition, pneumothorax is more likely to be refractory in these cases; initiation of nintedanib treatment and follow‐up should be considered carefully.

## Graphical abstract

We observed the development of refractory spontaneous pneumothorax during nintedanib therapy in two patients with systemic sclerosis and interstitial lung disease (SSc‐ILD) and impaired lung function. Nintedanib use for SSc‐ILD, an extensive disease, may therefore increase the risk of pneumothorax. In addition, pneumothorax is more likely to be refractory in these cases; initiation of nintedanib treatment and follow‐up should be considered carefully.

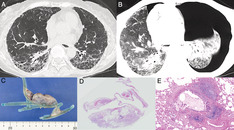

## Introduction

Systemic sclerosis (SSc) is a systemic connective tissue disease characterized by a combination of fibrosis of the skin and various organs, peripheral circulatory disturbances, and autoantibody production [1]. Functional disorders of the lungs, gastrointestinal tract, and heart may be present during the course of the disease. Interstitial lung disease (ILD) is the most common organ lesion associated with SSc, and it is also the most common cause of SSc‐related death, accounting for approximately 30% of deaths due to SSc [2, 3]. Pulmonary infection is the leading cause of death in patients with respiratory failure due to SSc‐ILD progression, followed by pneumothorax, lung cancer, and cardiopulmonary dysfunction with secondary pulmonary hypertension [4]. In the SENSCIS trial, nintedanib, an antifibrotic drug recently approved for the treatment of SSc‐ILD, significantly inhibited forced vital capacity (FVC) decline compared to placebo in patients with SSc‐ILD; measurements were obtained annually. A double‐blind clinical trial investigating the safety of nintedanib reported no pneumothorax in patients with SSc‐ILD [5]. However, patients in the SENSICS trial had a mean FVC of 2500 mL (73% of predicted), which indicates relatively good lung function. Nevertheless, the safety of nintedanib use in patients with impaired lung function is not clear. Here, we report on two patients with SSc‐ILD and impaired lung function, who developed refractory spontaneous pneumothorax during nintedanib therapy.

## Case Report

### Case 1

**Figure 1:**
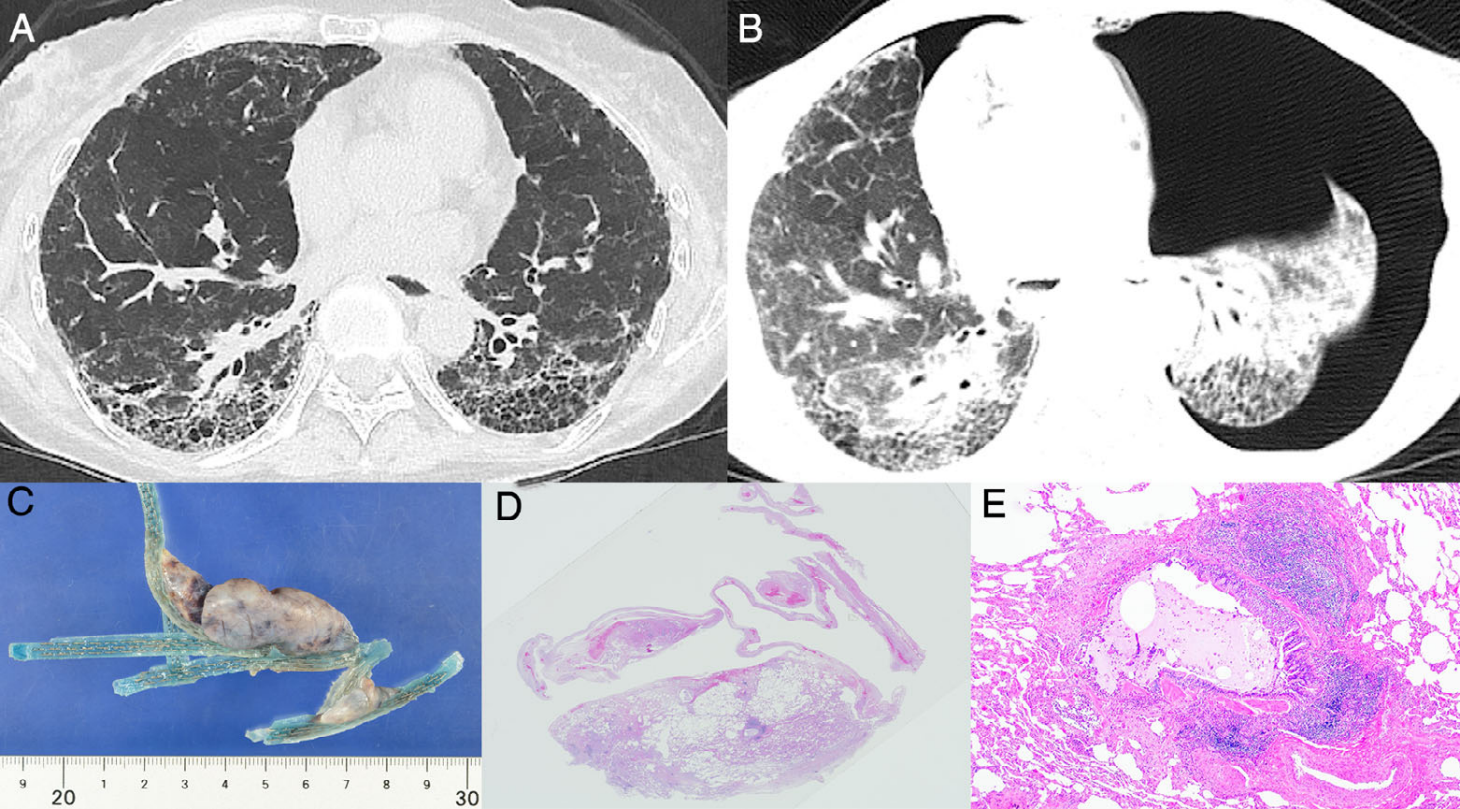
(A) Chest computed tomography showing ground‐glass opacities and honeycombing distributed dorsally in the lungs bilaterally. (B) The left lung is highly collapsed, and the right lung is mildly collapsed. (C–E) Pathological findings. (C) Macroscopic image of surgical specimens. (D) A bulla, an emphysematous cyst localized in the lungs, and a bleb with emphysematous cyst changes in the pleura (upper fragmentation); haematoxylin and eosin staining, loupe view. (E) Lymphocytes and plasmacytoid infiltrates in the peribronchial area with possible chronic inflammation associated with collagen disease; haematoxylin and eosin staining 10×.

A 62‐year‐old woman was referred to our hospital for a follow‐up evaluation of ILD. She was diagnosed with SSc at the age of 50 years, and had been treated with the immunosuppressant tacrolimus since the time of diagnosis. High‐resolution chest computed tomography showed ILD in 36.6% of the lung fields; the method of measurement was defined in a previous study [6] (Fig. 1A). Pulmonary function tests revealed an FVC of 1050 mL (43.6% of predicted) and a diffusing capacity for carbon monoxide (DLCO) of 47.8%; she had an elevated Sialylated carbohydrate antigen Krebs von den Lungen‐6 (KL‐6) level of 673 U/mL. A year later, she was started on 150 mg of nintedanib twice daily, because her FVC had declined by 140 mL and was predicted to decline further over time. No gastrointestinal symptoms such as diarrhoea or nausea were observed after starting nintedanib. Fourteen weeks after nintedanib initiation, she visited the emergency room due to dyspnoea and was diagnosed with pneumothorax (Fig. 1B). She was admitted to the hospital, nintedanib was stopped, and a chest tube was inserted. Thoracic drainage continued for three weeks, but the air leak persisted and the lung did not expand. Four weeks after admission, thoracoscopic bullectomy was performed despite the high surgical risk due to low lung function (Fig. 1C–E). After the operation, the air leak disappeared; the chest tube was removed two weeks after surgery.

### Case 2

**Figure 2:**
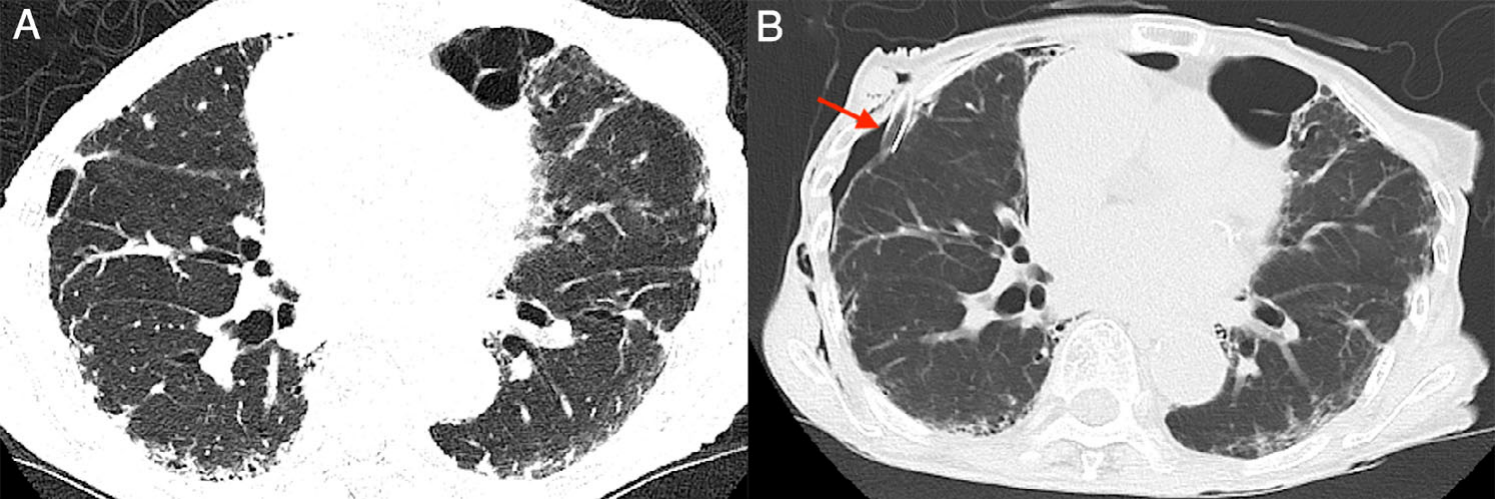
(A) Chest computed tomography showing subpleural interstitial shadows in the lungs. (B) A tube is inserted in the right thoracic cavity (red arrow), and mild subcutaneous emphysema is observed.

An 81‐year‐old woman was referred to our hospital for the treatment of interstitial pneumonia. History‐taking revealed that she was diagnosed with SSc at the age of 70 years; although she had fingertip ulcerations, she was not being treated with steroids or immunosuppressants. ILD was observed in 20% of the lung fields (Fig. 2A). Pulmonary function tests revealed an FVC of 1220 mL (65.2% of predicted) and a DLCO of 84.4%; she had an elevated KL‐6 level of 613 U/mL. Her FVC had declined by 220 mL a year later, so it was expected to decline further in the future. Therefore, 150 mg of nintedanib twice daily was started. Two weeks after starting nintedanib, the dose was reduced to 100 mg twice daily due to nausea. She continued the medication at this reduced dosage without adverse effects; however, 16 weeks after the start of treatment, she was admitted to an emergency department for dyspnoea and diagnosed with pneumothorax (Fig. 2B). She was admitted to the hospital, nintedanib was stopped, and a chest tube was inserted. As surgical treatment was not considered possible, pleural attachments (autologous blood, 50% glucose, 300 mg of minocycline, and 5 Klinische Einheit (KE) of picibanil) were performed weekly. The chest tube was removed seven weeks after admission.

## Discussion

We treated two patients with impaired lung function due to SSc‐ILD with nintedanib; both developed refractory spontaneous pneumothorax requiring prolonged drainage, pleurodesis, and surgery. It is necessary to consider that pneumothorax may occur during nintedanib treatment for low lung function in SSc‐ILD cases; if pneumothorax develops, it may be intractable.

In patients with severe lung dysfunction, the reversibility of ILD lesions is poor; in addition, the use of drugs with immunosuppressive effects may increase the risk of infection and death. Therapy with these drugs is therefore not recommended [4]. In elderly patients with severely deteriorated lung function (as in the present cases) who are unsuitable for lung transplantation, ILD progress is considered to be an extensive disease and the prognosis is poor [6]. Patients in the SENSCIS study, which showed that nintedanib reduced the annual FVC decline in patients with SSc‐ILD, had relatively good lung function [5]. There is currently no evidence that nintedanib, which has no immunosuppressive effect due to its mechanism of action, has any benefit in the treatment of patients with severe lung dysfunction. Therefore, it is necessary to evaluate the risks and benefits when administering nintedanib to patients with highly impaired lung function.

Pneumothorax was not reported with nintedanib use in the SENSCIS trial among patients with SSc‐ILD; it was also not reported in more than 5% of cases in the INPULSIS trial among patients with idiopathic pulmonary fibrosis (IPF) [5, 7]. In a post‐marketing survey in Japan, the frequency of pneumothorax in patients with IPF treated with nintedanib was also low at 0.33% [8]. However, both clinical trials were conducted in patients with relatively good lung function, with an average FVC of more than 70% of predicted. The two patients described here did not meet the exclusion criteria and one inclusion criteria (duration of less than seven years after onset of the first non‐Raynaud's symptom attributable to SSc) of the SENSCIS trial, but fulfilled all the other inclusion criteria (ILD ≥10%, %FVC ≥40%, and 30% ≤ %DLCO ≤ 89%). Patients with similar disease phenotypes were not included in the SENSCIS trial, and the safety profile of nintedanib is currently largely unknown in this population.

In IPF cases, spontaneous pneumothorax is a known and relatively frequent complication in IPF [9], and the risk of pneumothorax increases as the disease progresses [10]. Although spontaneous pneumothorax is a rare complication of SSc, it is also a likely cause of death in cases of advanced or severe ILD [4]. Nintedanib is a small‐molecule tyrosine kinase inhibitor against vascular endothelial growth factor (VEGF) receptor (VEGFR), platelet‐derived growth factor receptor‐alpha (PDGFRα), and fibroblast growth factor receptor (FGFR). Similarly, bevacizumab, which has an anti‐VEGF effect, has been shown to cause ischaemic changes and perforation of the lung tissue due to its anti‐VEGF action [11]. As the lungs of patients with progressed interstitial pneumonia have diminished elastic fibres and fragile lung tissue structure, the anti‐VEGF effect of nintedanib may have caused pneumothorax in the present patients who showed notable fibrosis. In addition, VEGFR, PDGFRα, and FGFR are involved in wound healing; therefore, delayed wound healing may occur in patients taking nintedanib. There were no reports on protracted wound healing in the nintedanib group of the SENSCIS study, and there was no difference in the occurrence of skin ulcers as an adverse event between the nintedanib and placebo groups (18.4% and 17.4%, respectively) [5]. However, a Japanese subgroup analysis of the SENSCIS revealed a trend towards increased prevalence of digital ulcers in the nintedanib than in the placebo group [12]. There may be racial differences in impaired wound healing with nintedanib.

The half‐life from the steady state of 300 mg of nintedanib twice daily is 27.5 h [13]; consequently, blood levels of nintedanib are expected to decline gradually after discontinuation. Therefore, the most likely reason for the refractory pneumothorax in these two cases was the inability of the lungs to reinflate sufficiently after thoracic drainage due to decreased lung compliance from interstitial pneumonia.

In summary, nintedanib treatment for SSc‐ILD, which is classified as an extensive disease, possibly increases the risk of refractory pneumothorax. However, this report only describes two patients, and this observation may have been a coincidence. Cumulative evidence from future cases is needed for evaluating the potential risk of this serious complication.

### Disclosure Statement

Appropriate written informed consent was obtained for publication of this case report and accompanying images.

### Author Contribution Statement

Conceptualization: Toshiyuki Sumi. Investigation: Toshiyuki Sumi, Hirofumi Uehara, Makoto Tada, Yoshiko Keira, Koki Kamada, Naoki Shijubou, Yuichi Yamada. Writing—original draft: Toshiyuki Sumi. Writing—review and editing: Hisashi Nakata, Yuji Mori, Hirofumi Chiba.
